# Supplementary material for: Maternal and neonatal outcomes of antihypertensive treatment in pregnancy: A retrospective cohort study
Source: PLoS One. 2022 May 16;17(5):e0268284. doi: 10.1371/journal.pone.0268284 (PMC9109931; doi:10.1371/journal.pone.0268284)
Supplement: S1 File — (DOCX) [file pone.0268284.s001.docx]

**Supporting Information**

[**S1 Fig. Distribution of the probability of use of each medication (i.e., propensity score) across medication group 2**](#_Toc78979665)

[**S2 Fig. Sensitivity Analysis: Maternal and infant outcomes in relation to antihypertensive medication exposure in pregnancy, restricted to women with chronic hypertension*. 3**](#_Toc78979666)

[**S3 Fig. Sensitivity analysis: maternal and infant outcomes in relation to antihypertensive medication exposure in pregnancy, after excluding women with pre-gestational diabetes*. 5**](#_Toc78979667)

[**S4 Fig. Maternal and infant outcomes in relation to antihypertensive medication exposure in pregnancy, by treatment history: new users*. 7**](#_Toc78979668)

[**S5 Fig. Maternal and infant outcomes in relation to antihypertensive medication exposure in pregnancy, by treatment history: women with prior antihypertensive medication use*. 8**](#_Toc78979669)

[**S1 Table. Study variable definitions. 10**](#_Toc78979670)

[**S2 Table. Covariates included in propensity score models predicting type of antihypertensive medication used. 18**](#_Toc78979671)

[**S3 Table. Covariates included in model for inverse probability of censoring weights*. 19**](#_Toc78979672)

[**S4 Table. Baseline characteristics of the population, overall and by medication category, before inverse probability of treatment weighting (expanded)*. 20**](#_Toc78979673)

[**S5 Table. Baseline characteristics by medication exposure category, after inverse probability of treatment weighting*. 23**](#_Toc78979674)

[**S6 Table. Distribution of analytic weights. 27**](#_Toc78979675)

| 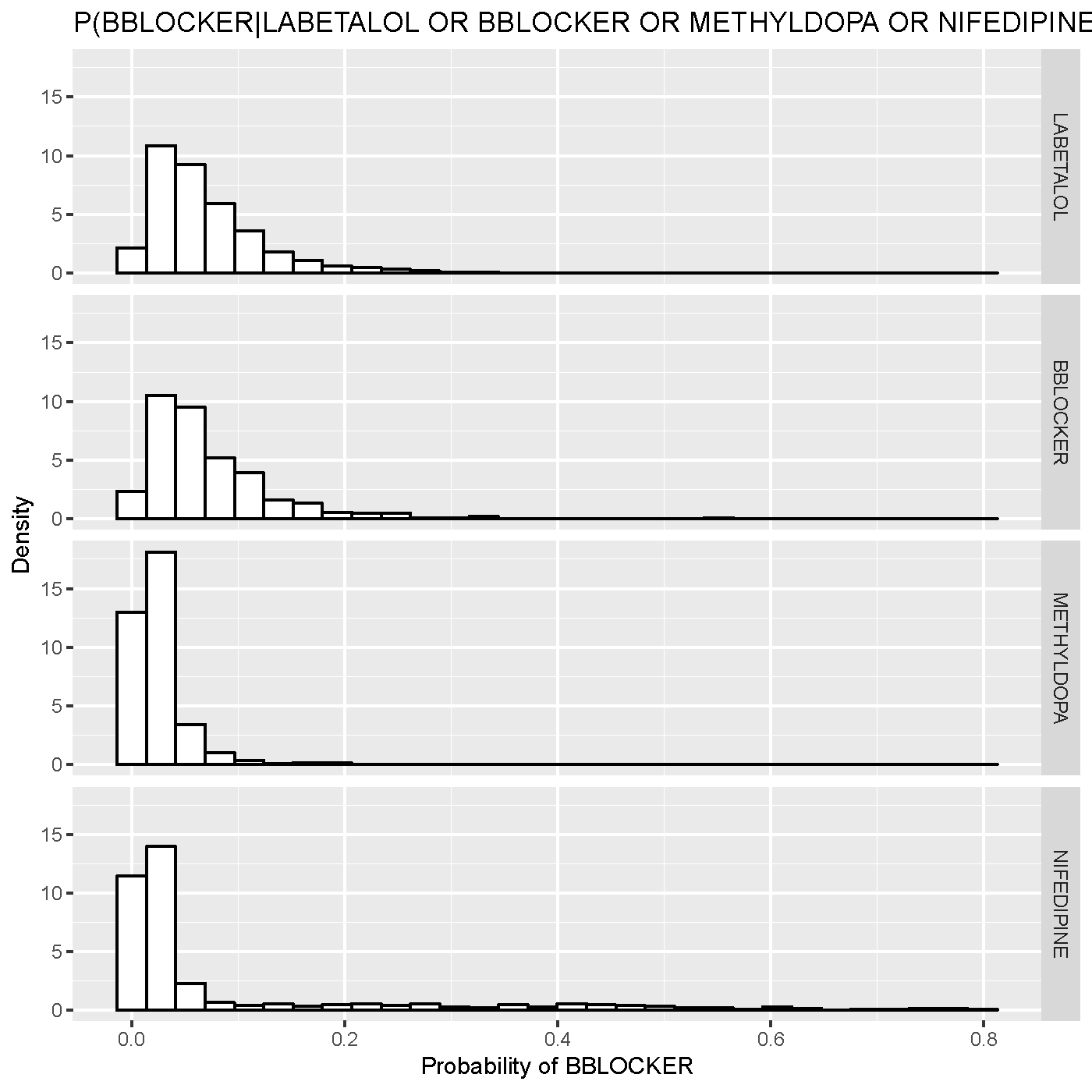S1 Fig. Distribution of the probability of use of each medication (i.e., propensity score) across medication group**.** The distribution of the estimated probability of receiving labetalol (a), methyldopa (b), other beta blockers (c), and nifedipine (d) for women assigned to labetalol, beta blockers, methyldopa, and nifedipine, from top to bottom given all measured confounders. | |
| --- | --- |
| (a) | (c) |
|  |  |
| (b) | (d) |

# S2 Fig. Sensitivity Analysis: Maternal and infant outcomes in relation to antihypertensive medication exposure in pregnancy, restricted to women with chronic hypertension.^a^


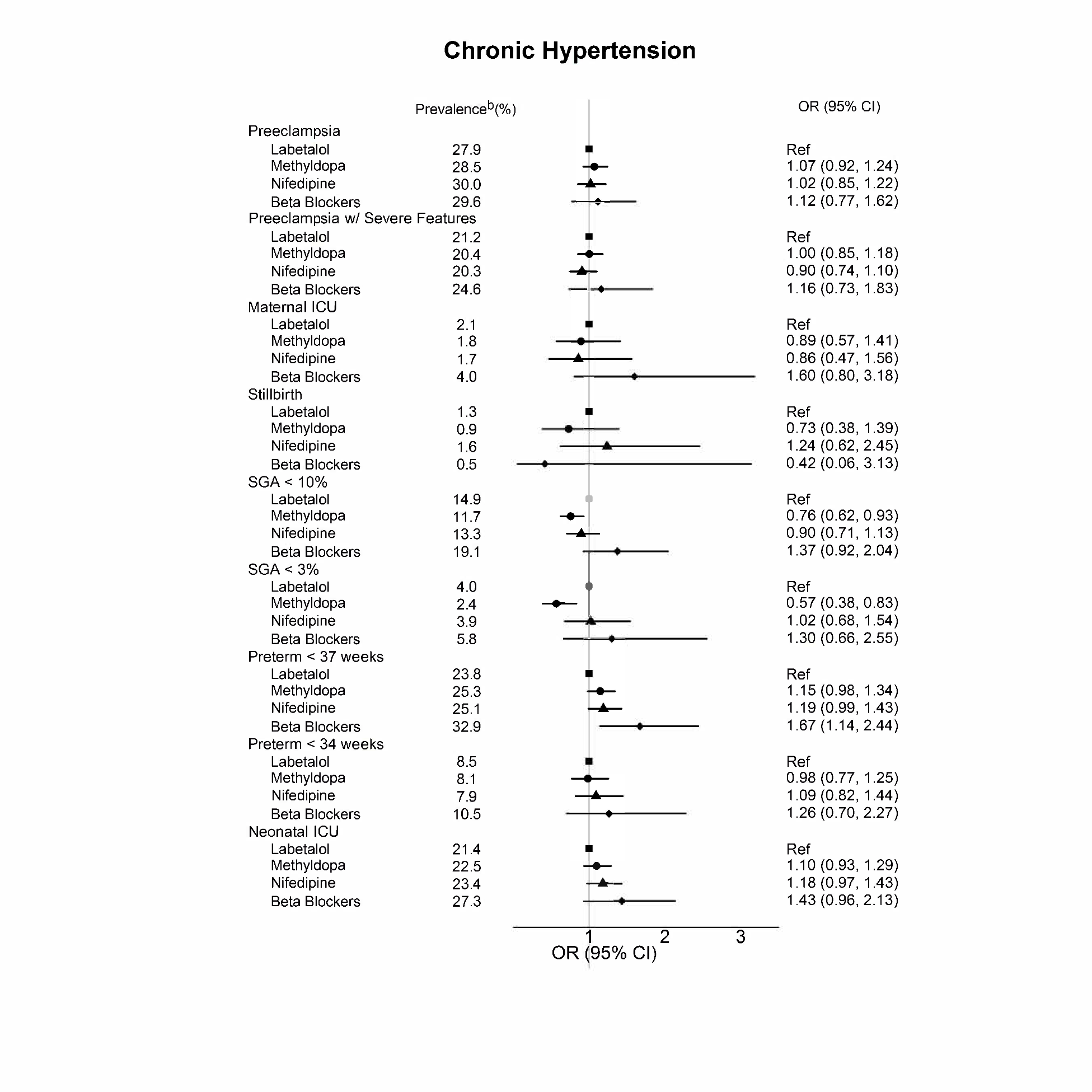


Abbreviations: OR, odds ratio; CI, confidence interval; SGA, small for gestational age; ICU, intensive care unit.

^a^Labetalol is the referent group for all comparisons. The population for different outcomes differs slightly because pregnancy losses (stillbirth or termination) were not included in the denominator for the outcomes of SGA, preterm delivery, or neonatal ICU admission. For most outcomes, the total N is 5513, for SGA it is 5422, and for preterm delivery and NICU admission it is 5450.

^b^Weighted prevalence in the subgroup after inverse probability of treatment weighting using unstabilized weights.

# S3 Fig. Sensitivity analysis: maternal and infant outcomes in relation to antihypertensive medication exposure in pregnancy, after excluding women with pre-gestational diabetes.^a^


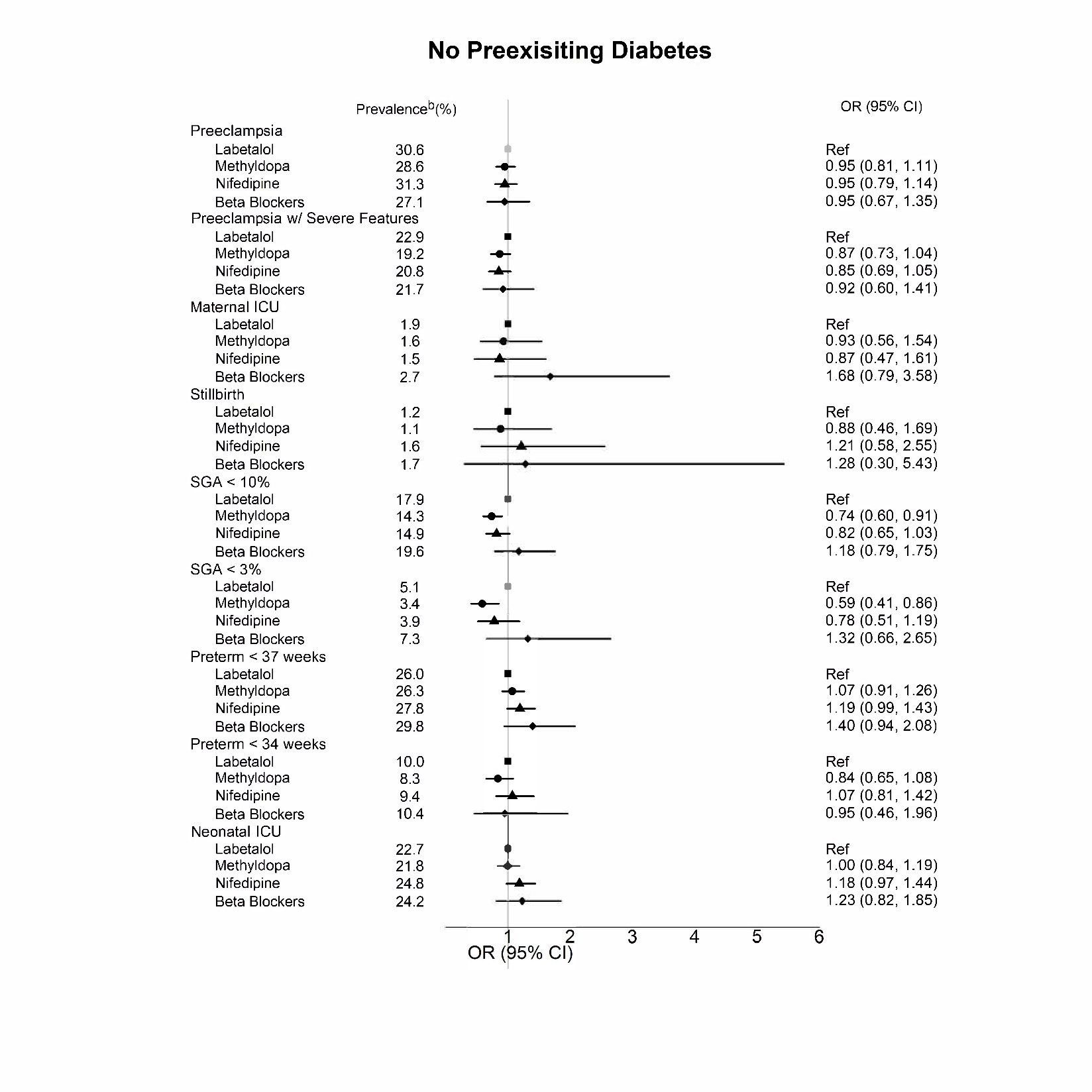


Abbreviations: OR, odds ratio; CI, confidence interval; SGA, small for gestational age; ICU, intensive care unit.

^a^Labetalol is the referent group for all comparisons. The population for different outcomes differs slightly because pregnancy losses (stillbirth or termination) were not included in the denominator for the outcomes of SGA, preterm delivery, or neonatal ICU admission. For most outcomes, the total N is 5113, for SGA it is 5023, and for preterm delivery and NICU admission it is 5052.

^b^Weighted prevalence in the subgroup after inverse probability of treatment weighting using unstabilized weights.

# S4 Fig. Maternal and infant outcomes in relation to antihypertensive medication exposure in pregnancy, by treatment history: new users.^a^


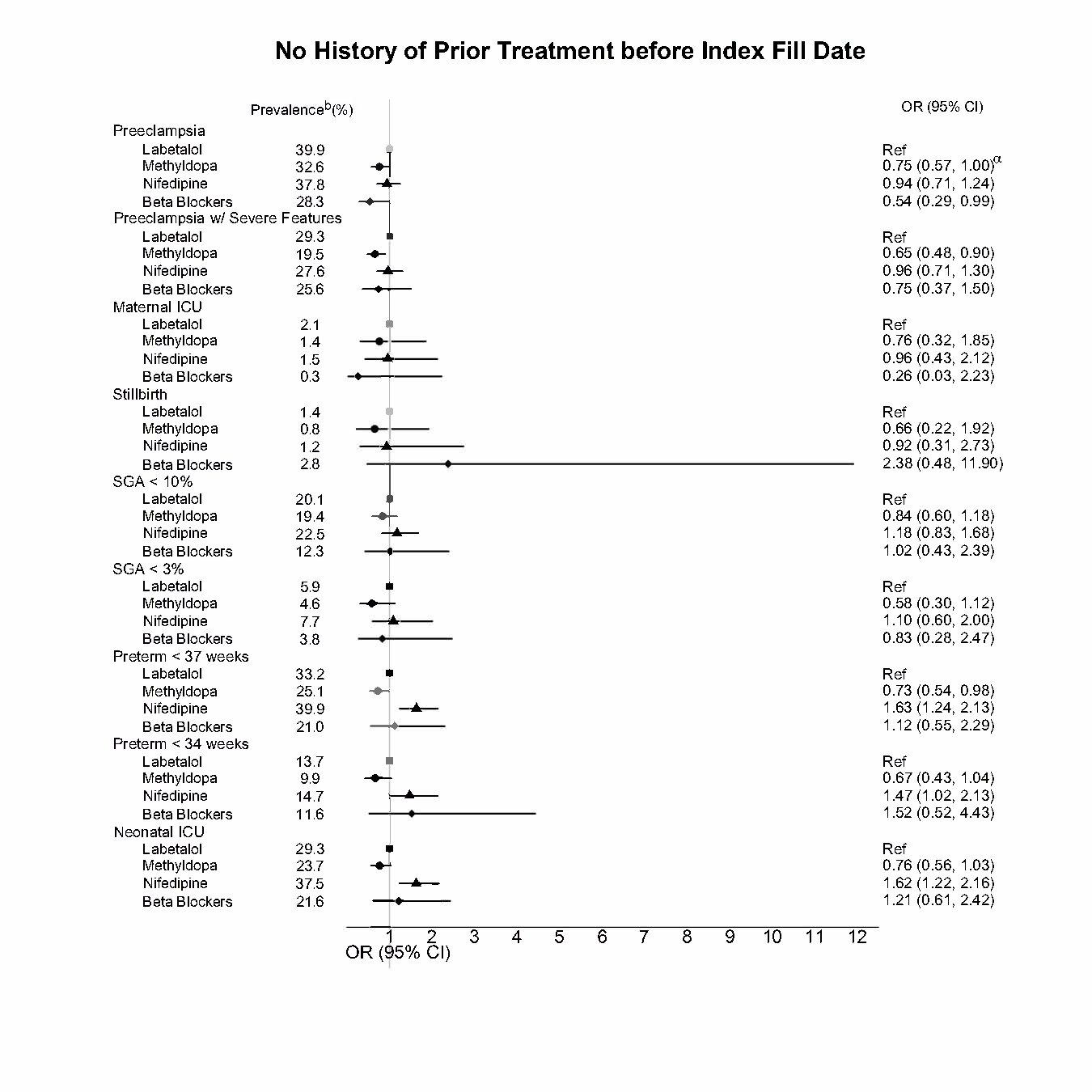


Abbreviations: OR, odds ratio; CI, confidence interval; SGA, small for gestational age; ICU, intensive care unit.

^a^Labetalol is the referent group for all comparisons. The population for different outcomes differs because pregnancy losses (stillbirth or termination) were not included in the denominator for the outcomes of SGA, preterm delivery, or neonatal ICU admission. For most outcomes, the total N is 2166, for SGA it is 2122, and for preterm delivery and NICU admission it is 2136.
^b^Weighted prevalence in the subgroup after inverse probability of treatment weighting using unstabilized weights.

α: significant at the level of p<0.05

# S5 Fig. Maternal and infant outcomes in relation to antihypertensive medication exposure in pregnancy, by treatment history: women with prior antihypertensive medication use.^a^


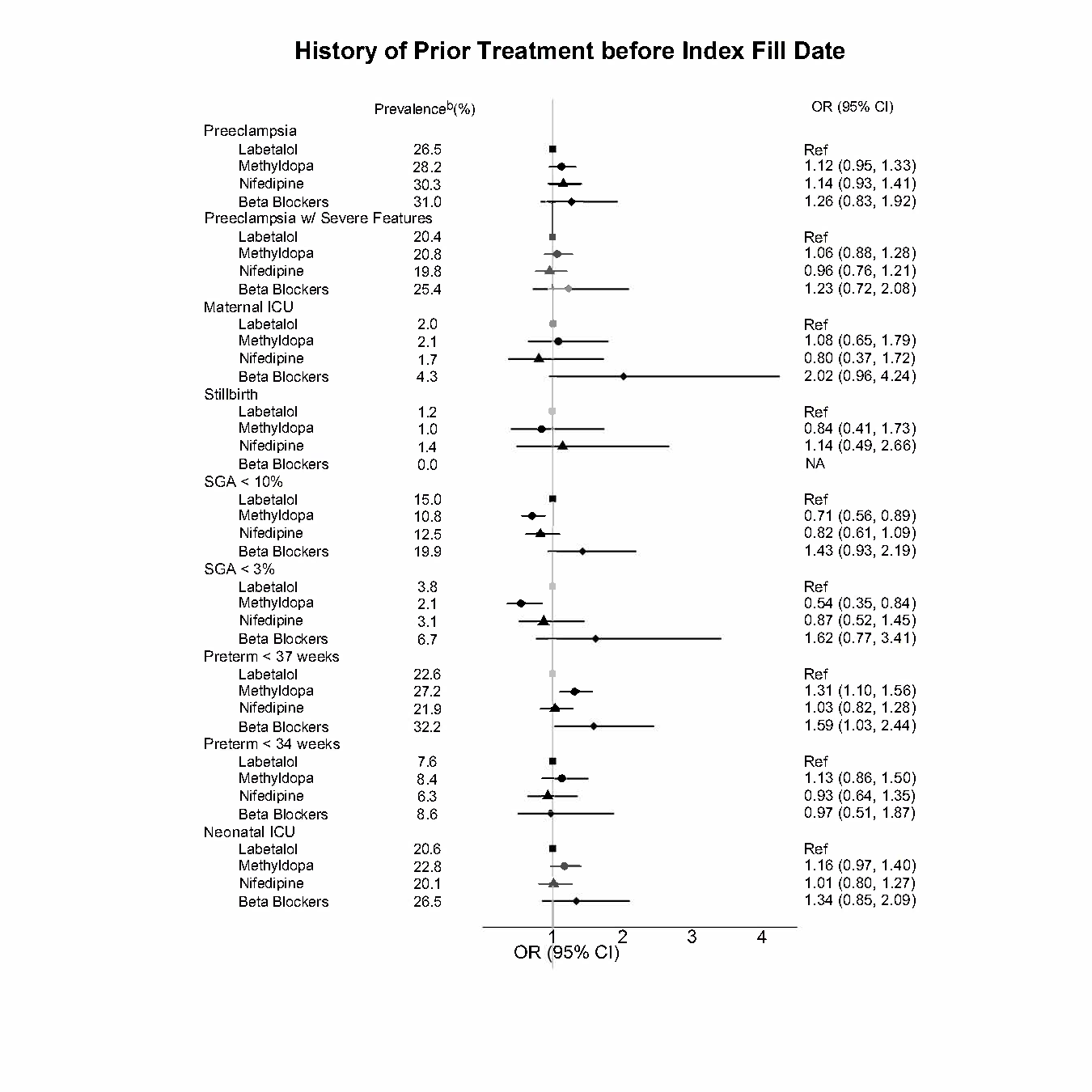


Abbreviations: OR, odds ratio; CI, confidence interval; SGA, small for gestational age; ICU, intensive care unit.

^a^Labetalol is the referent group for all comparisons. The population for different outcomes differs slightly because pregnancy losses (stillbirth or termination) were not included in the denominator for the outcomes of SGA, preterm delivery, or neonatal ICU admission. For most outcomes, the total N is 4180, for SGA it is 4118, and for preterm delivery and NICU admission it is 4136.

^b^Weighted prevalence in the subgroup after inverse probability of treatment weighting using unstabilized weights.

# S1 Table. Study variable definitions.

| **Inclusion criteria** |  |
| --- | --- |
| Criteria for identifying hypertension during pregnancy | 1. Two high BP values (≥140/90) within 30 days during pregnancy, or 2. One or more fills for an antihypertensive medication^a^ during the 120 days prior to pregnancy along with ≥ 1 hypertension diagnosis code (see below) during the 1 year prior to pregnancy or the first 20 weeks of pregnancy, or 3. 1 or more BP values ≥140/90 during pregnancy along with at least 1 diagnosis code for hypertension and 1 antihypertensive medication fill^a^ within 7 days of the elevated BP.   ^a^For this algorithm, a comprehensive list of antihypertensive medications was used, not just those eligible to be the index fill for this analysis.  We have published more information about this definition and our rationale here:  Chen L, Shortreed SM, Easterling T, Cheetham TC, Reynolds K, Avalos LA, Kamineni A, Holt V, Neugebauer R, Akosile M, Nance N, Bider-Canfield Z, Walker RL, Badon SE, Dublin S. [Identifying hypertension in pregnancy using electronic medical records: The importance of blood pressure values.](https://www-ncbi-nlm-nih-gov.offcampus.lib.washington.edu/pubmed/31954339) Pregnancy Hypertens. 2020 Jan;19:112-118. doi: 10.1016/j.preghy.2020.01.001. Epub 2020 Jan 3. |
| Hypertension diagnosis codes | ICD-9 codes 401-405, 437.2, 642.00-642.04, 642.10-642.14, 642.20-642.24, 642.30-642.34, 642.70-642.74, 642.9, 760.0 |
| Antihypertensive medications: all | Electronic pharmacy data were queried using the list below to identify antihypertensive medication fills for the purpose of applying the definition of hypertension above. Not all of the medications below were actually found in the data.  Acebutolol  Acetazolamide  Aliskiren  Amiloride  Amlodipine  Atacand  Atenolol  Benazepril  Betaxalol  Bisoprolol  Bumetanide  Candesartan  Captopril  Carteolol  Carvedilol  Chlorothiazide  Chlorthalidone  Clonidine  Deserpidine  Diltiazem  Doxazosin  Enalapril  Enalaprilat  Eprosartan  Esmolol  Ethacrynate  Felodipine  Fosinopril  Furosemide  Guanabenz  Guanfacine  Hydralazine  Hydrochlorothiazide  Indapamide  Isradipine  Labetalol  Levobunolol  Lisinopril  Losartan  Mecamylamine  Methylclothiazide  Methyldopa  Metolazone  Metoprolol  Metripranolol  Minoxidil  Moexiil  Monopril  Nadolol  Nebivolol  Nicardipine  Nifedipine  Nimodipine  Nisoldipine  Olmesartan  Penbutolol  Phenoxybenzamine  Pindolol  Prazosin  Propranolol  Quinapril  Ramipril  Reserpine  Sotalol  Spironolactone  Telmisartan  Terazosin  Timolol  Torsemide  Trandolapril  Triamterene  Valsartan  Verapamil |
| Antihypertensive medications that could qualify as the index fill | Only oral medications were included.  Medications of interest: labetalol, methyldopa, nifedipine, and other β-blockers  Other β-blockers:  Acebutolol  Atenolol  Betaxalol  Bisoprolol  Carteolol  Carvedilol  Esmolol  Levobunolol  Metoprolol  Metripranolol  Nadolol  Nebivolol  Penbutolol  Pindolol  Propranolol  Sotalol  Timolol |
| **Exclusion criteria** |  |
| Exposure to teratogenic medication | Any fills from 120 days prior to pregnancy through delivery; oral forms only:   - Isotretinoin - Bexarotene - Acitretin - Methotrexate - Mycophenolate - Thalidomide - Warfarin - Lithium - Azathioprine - Leflunomide - Valproic Acid - Sodium Valproate - Divalproex - Carbamazepine - Phenytoin (Fosphenytoin) - Tacrolimus - Sirolimus - Cyclosporine   Any fills from 90 days prior to pregnancy up through 20 weeks’ gestation, either oral or vaginal form:   - Misoprostol |
| Multiple gestation | ICD-9 codes: 651.0, 651.00, 651.01, 651.03, 651.1, 651.10, 651.11, 651.13, 651.2, 651.20, 651.21, 651.23, 651.3, 651.30, 651.31, 651.33, 651.4, 651.40, 651.41, 651.43, 651.5, 651.50, 651.51, 651.53, 651.6, 651.60, 651.61, 651.63, 651.7, 651.70, 651.71, 651.73, 651.8, 651.80, 651.81, 651.83, 651.9, 651.90, 651.91, 651.93, 652.6, 652.60, 652.61, 652.63, 761.5, V27.2 -V27.7, V91  Or designated as multiple gestation in a site’s clinical pregnancy data. |
| Certain serious chronic heart diseases | ICD-9 codes 394.0, 412, 416.0, 416.2, 416.8, 425.1, 426.82, 429.4, 648.51, 648.53 recorded from 1 year prior to start of pregnancy through delivery |
| Certain serious chronic kidney diseases | ICD-9 codes 403.01, 585.4-585.69, 586, V45.11 recorded from 1 year prior to start of pregnancy through delivery |
| Sickle cell anemia | ICD-9 code 282.6X |
| Systemic lupus erythematosus | ICD-9 code 710.0 |
| Systemic sclerosis (scleroderma) | ICD-9 code 710.1X, 517.2X, 583.9X |
| **Outcomes** | |
| Preeclampsia/  eclampsia | An inpatient ICD-9 diagnosis code of: 642.4, 642.5, 642.6, 642.7 |
| Preeclampsia with severe features | Meets criteria for pre-eclampsia and at least one of the following:   1. 2 BP values of ≥160/110 at least 4 hours apart 2. Abnormal laboratory values: creatinine > 1.1, platelets < 100,000/microliter, AST ≥ 2x upper limit of normal, or  ALT ≥ 2 x upper limit of normal 3. Diagnosis code for pulmonary edema, ICD-9 code 514 or 518.4 4. Diagnosis of eclampsia, ICD-9 code 642.6 |
| Stillbirth or termination at ≥ 20 weeks gestation | ICD-9 codes used to identify potential cases to undergo chart review and/or linkage to fetal death certificates:  Stillbirth codes:  646.01, 656.4, 656.40, 656.41, 656.43, 768.0, 768.1, V27.1, V27.4, V27.7, V27.3, V27.6, V32, V32.0, V32.00, V32.01, V32.1, V32.2, V35, V35.0, V35.00, V35.01, V35.1, V35.2, V36, V36.0, V36.00, V36.01, V36.1, V36.2  Termination diagnosis codes:  635, 635.0, 635.00, 635.01, 635.02, 635.1, 635.10, 635.11, 635.12, 635.2, 635.20, 635.21, 635.22, 635.3, 635.30, 635.31, 635.32, 635.4, 635.40, 635.41, 635.42, 635.5, 635.50, 635.51, 635.52, 635.6, 635.60, 635.61, 635.62, 635.7, 635.70, 635.71, 635.72, 635.8, 635.80, 635.81, 635.82, 635.9, 635.90, 635.91, 635.92, 636, 636.0, 636.00, 636.01, 636.02, 636.1, 636.10, 636.11, 636.12, 636.2, 636.20, 636.21, 636.22, 636.3, 636.30, 636.31, 636.32, 636.4, 636.40, 636.41, 636.42, 636.5, 636.50, 636.51, 636.52, 636.6, 636.60, 636.61, 636.62, 636.7, 636.70, 636.71, 636.72, 636.8, 636.80, 636.81, 636.82, 636.9, 636.90, 636.91, 636.92, 637.xx, 779.6  Termination procedure codes:  01964, 01966, 59830, 59840, 59841, 59850, 59851, 59852, 59855, 59856, 59857, 69.01, 69.51, 74.91, 75.0, S0199, S2260, S2262, S2265, S2266, S2267  Miscarriage codes (cases were selected for medical record review and possible inclusion if gestational age was recorded in the EHR as being ≥ 20 weeks):  *ICD-9 diagnosis codes:* 631, 632, 634, 640.01, 640.81, 640.91  *Procedure codes:* 59812, 59820, 59821 |
| **Covariates** | |
| Chronic hypertension | (Adaptation of the criteria used above to define hypertension, focusing on the time period from 2 years prior to the start of pregnancy through 20 weeks gestation):   1. 2 BPs ≥ 140/90 within 30 days, or 2. ≥1 hypertension diagnosis code and ≥1 antihypertensive medication fill within 12 months of each other, or 3. 1 BP ≥ 140/90 along with 1+ hypertension diagnosis code(s) and 1+ medication fill(s) within 7 days of the elevated BP |
| Gestational hypertension | Meets criteria for hypertension in pregnancy but does not meet any of the criteria above for chronic hypertension |
| Prior antihypertensive medication use | Categorized as no prior use, continuous use up to the index fill, or prior use with a gap.  No prior use was defined as no fills for an antihypertensive medication during pregnancy before the index fill or in the two years prior to pregnancy.  Continuous use: we estimated a run-out date for the antihypertensive medication fill prior to the index date assuming 80% adherence. If this run-out date was on or after the index fill date, then use was considered continuous. If a woman had multiple prescriptions for antihypertensive medications prior to the index fill date, then the latest run-out date was used.  Prior use with a gap: if a woman had prior antihypertensive medication fills before the index date but run-out dates (assuming 80% adherence) were before the date of the index fill, she was considered to have a gap in use. |
| Diabetes | 1) ≥1 inpatient ICD-9-CM codes 250.XX (all inclusive) or 648.0X (all inclusive) or 648.8X (all inclusive)  OR  2)  ≥2 outpatient or emergency department ICD-9-CM codes 250.XX (all inclusive) or 648.0X (all inclusive) or 648.8X (all inclusive) that occurred on different dates |
| Depression | Two outpatient diagnosis codes, or one inpatient code, or one outpatient code and one dispensing for an antidepressant.  ICD-9-CM diagnosis codes: 311.XX  See below for a list of antidepressant names. |
| Statin medications | Atorvastatin Cerivastatin Fluvastatin  Lovastatin  Pravastatin Rosuvastatin Simvastatin  Pitavastatin |
| Benzodiazepine medications | Alprazolam  Chlordiazepoxide  Clonazepam  Clorazepate  Diazepam  Estazolam  Flurazepam  Lorazepam  Oxazepam  Quazepam  Temazepam  Triazolam |
| Antidepressant medications | Amitriptyline  Amoxapine  Atomoxetine  Bupropion  Citalopram  Clomipramine  Desipramine  Desvenlafaxine  Doxepin  Duloxetine  Escitalopram  Fluoxetine  Fluvoxamine  Isocarboxazid  Maprotiline  Milnacipran  Mirtazapine  Nefazodone  Nortriptyline  Paroxetine  Phenelzine  Protriptyline  Selegiline  Sertraline  Tranylcypromine  Trazodone  Trimipramine  Venlafaxine  Vilazodone |
| Diabetes medications | Insulins  Lantus  Glargine  Regular  NPH  Aspart  Lispro  Detemir  Glulisine  Oral medications  Metformin  Acetohexamide Chlorpropamide Glimepiride Glipizide Glyburide Tolazamide Tolbutamide  Gliclazide  Pioglitazone Rosiglitazone Acarbose Miglitol Voglibose Sitagliptin Saxagliptin Linagliptin Nateglinide Repaglinide Exenatide  Pramlintide |
| Anticonvulsant medications | Phenytoin (Fosphenytoin)  Phenobarbital (Mephobarbital)  Primidone  Ethosuximide  Gabapentin  Lamotrigine  Levetiracetam  Oxcarbazepine  Pregabalin  Tiagabine  Topiramate  Zonisamide |

# S2 Table. Covariates included in propensity score models predicting type of antihypertensive medication used.

| **Variables** |
| --- |
| Maternal age at delivery (grouped as <30, 30-34, 35-39 or ≥ 40 years) |
| Maternal race/ethnicity |
| Year of delivery (grouped as 2005-2008, 2009-2010, 2011-2012, 2013-2014) |
| Kaiser Permanente region (Northern California, Southern California or Washington) |
| Medicaid insurance |
| Tobacco use during pregnancy |
| Depression |
| Pregestational diabetes |
| Type of hypertension (chronic vs. gestational) |
| Gestational age at time of index fill (modeled as a linear spline with knots at 140 and 210 days gestation) |
| Prior history of antihypertensive medication use (no use, prior use with continuous use up to index date, prior use with a gap before index date) |
| History of ACEI/ARB use |
| History of thiazide diuretic use |
| Use of antidiabetic medications |
| Statin use |
| Benzodiazepine use |
| Anticonvulsant use |
| Antidepressant use |
| BP value closest to and prior to index fill (systolic and diastolic BP values modeled separately, entered into model as continuous variables using linear splines, with knots at 140 mm Hg for systolic BP and 90 mm Hg for diastolic BP) |
| History of one or more BP values ≥ 160/110 |

Abbreviations: ACEI, angiotensin converting enzyme inhibitor; ARB, angiotensin receptor blocker; BP, blood pressure.

# S3 Table. Covariates included in model for inverse probability of censoring weights.^a^

| **Variables** |
| --- |
| Maternal age at delivery |
| Maternal race/ethnicity |
| Kaiser Permanente region (Northern California, Southern California or Washington) |
| Tobacco use during pregnancy |
| Gestational age at time of index fill |
| Type of antihypertensive medication used (4 levels) |
| Pre-gestational diabetes |
| BP value closest to and prior to index fill (systolic and diastolic BP values, modeled separately) |
| History of one or more BP values ≥ 160/110 |

Abbreviations: BP, blood pressure.

^a^For analyses that excluded stillbirths and terminations due to competing risks.

# S4 Table. Baseline characteristics of the population, overall and by medication category, before inverse probability of treatment weighting (expanded).^a^

| **Characteristic** | **Overall**  **N=6346** | **Labetalol**  **N=3017** | **Methyldopa**  **N=1834** | **Nifedipine**  **N=1105** | **Other  β- blockers**  **N=390** |
| --- | --- | --- | --- | --- | --- |
| Maternal age, in years; mean(SD) | 33.6±5.3 | 33.5±5.2 | 33.9±5.3 | 33.2±5.6 | 33.8±5.2 |
| Parity,^b^ no. (%) |  |  |  |  |  |
| Nulliparous | 2369 (37.3) | 1109 (36.8) | 699 (38.1) | 422 (38.2) | 139 (35.6) |
| Parous | 3757 (59.2) | 1781 (59.0) | 1084 (59.1) | 652 (59.0) | 240 (61.5) |
| Missing | 220 (3.5) | 127 (4.2) | 51 (2.8) | 31 (2.8) | 11 (2.8) |
| Race/ethnicity, no. (%) |  |  |  |  |  |
| White, non-Hispanic | 2106 (33.2) | 1011 (33.5) | 551 (30.0) | 369 (33.4) | 175 (44.9) |
| Hispanic | 1882 (29.7) | 928 (30.8) | 604 (32.9) | 275 (24.9) | 75 (19.2) |
| Black, non-Hispanic | 956 (15.1) | 470 (15.6) | 242 (13.2) | 184 (16.7) | 60 (15.4) |
| Asian | 1323 (20.8) | 575 (19.1) | 414 (22.6) | 261 (23.6) | 73 (18.7) |
| Other | 46 (0.7) | 22 (0.7) | 11 (0.6) | 10 (0.9) | 3 (0.8) |
| Unknown | 33 (0.5) | 11 (0.4) | 12 (0.7) | 6 (0.5) | 4 (1.0) |
| Maternal BMI,^b^ no. (%) |  |  |  |  |  |
| Normal or underweight (< 25 kg/m^2^) | 889 (14.0) | 407 (13.5) | 253 (13.8) | 180 (16.3) | 49 (12.6) |
| Overweight (25-29.9 kg/m^2^) | 1377 (21.7) | 702 (23.3) | 358 (19.5) | 252 (22.8) | 65 (16.7) |
| Obese (≥ 30 kg/m^2^) | 3568 (56.2) | 1796 (59.5) | 968 (52.8) | 581 (52.6) | 223 (57.2) |
| Missing | 512 (8.1) | 112 (3.7) | 255 (13.9) | 92 (8.3) | 53 (13.6) |
| Tobacco use in pregnancy, no. (%) | 292 (4.6) | 150 (5.0) | 68 (3.7) | 53 (4.8) | 21 (5.4) |
| Chronic hypertension, no. (%) | 5513 (86.9) | 2550 (84.5) | 1693 (92.3) | 910 (82.4) | 360 (92.3) |
| Pre-gestational diabetes, no. (%) | 1233 (19.4) | 560 (18.6) | 355 (19.4) | 250 (22.6) | 68 (17.4) |
| Education,^b^ no. (%) |  |  |  |  |  |
| Less than college | 3350 (52.8) | 1588 (52.6) | 975 (53.2) | 570 (51.6) | 217 (55.6) |
| Completed college, or beyond | 2724 (42.9) | 1308 (43.4) | 782 (42.6) | 479 (43.3) | 155 (39.7) |
| Missing | 272 (4.3) | 121 (4.0) | 77 (4.2) | 56 (5.1) | 18 (4.6) |
| Timing of initiating prenatal care,^b^ no. (%) |  |  |  |  |  |
| First trimester^c^ | 5254 (82.8) | 2653 (87.9) | 1402 (76.4) | 922 (83.4) | 277 (71.0) |
| After first trimester | 423 (6.7) | 175 (5.8) | 145 (7.9) | 74 (6.7) | 29 (7.4) |
| Missing | 669 (10.5) | 189 (6.3) | 287 (15.6) | 109 (9.9) | 84 (21.5) |
| Weeks of gestation at index fill, mean±SD | 18.4±9.2 | 18.8±9.6 | 16.5±8.0 | 20.8±9.6 | 17.1±7.9 |
| Systolic BP before index fill, mm Hg; mean±SD | 140.3 (17.0) | 142.8 (17.2) | 138.7 (15.7) | 138.9 (17.0) | 132.8 (17.3) |
| Diastolic BP before index fill, mm Hg; mean±SD | 86.1 (12.0) | 88.0 (12.1) | 84.9 (10.9) | 84.9 (12.9) | 80.7 (11.9) |
| BP ≥ 160/110 before the start of this pregnancy, no. (%) | 1864 (29.4) | 901 (29.9) | 538 (29.3) | 309 (28.0) | 116 (29.7) |
| BP ≥ 160/110 during pregnancy before the index fill, no. (%) | 1180 (18.6) | 669 (22.2) | 270 (14.7) | 204 (18.5) | 37 (9.5) |
| Prior antihypertensive medication use, no. (%) |  |  |  |  |  |
| Prior use, continuous | 2341 (36.9) | 1018 (33.7) | 813 (44.3) | 348 (31.5) | 162 (41.5) |
| Prior use with a gap | 1839 (29.0) | 839 (27.8) | 577 (31.5) | 296 (26.8) | 127 (32.6) |
| No prior use | 2166 (34.1) | 1160 (38.4) | 444 (24.2) | 461 (41.7) | 101 (25.9) |
| Delivery year, no. (%) |  |  |  |  |  |
| 2005-2008 | 1657 (26.1) | 500 (16.6) | 704 (38.4) | 314 (28.4) | 139 (35.6) |
| 2009-2010 | 1551 (24.4) | 713 (23.6) | 489 (26.7) | 250 (22.6) | 99 (25.4) |
| 2011-2012 | 1584 (25.0) | 860 (28.5) | 348 (19.0) | 287 (26.0) | 89 (22.8) |
| 2013-2014 | 1554 (24.5) | 944 (31.3) | 293 (16.0) | 254 (23.0) | 63 (16.2) |

Abbreviations: BMI, body mass index; BP, blood pressure; SD, standard deviation.

^a^All characteristics measured prior to the index medication fill, except for delivery year.

^b^Covariates that are not in the propensity score model.

^c^Defined as first 90 days of pregnancy.

# S5 Table. Baseline characteristics by medication exposure category, after inverse probability of treatment weighting.^a^

| **Characteristic** | **Labetalol** | **Methyldopa** | **Nifedipine** | **Other beta blockers** | **SMD equivalent** |
| --- | --- | --- | --- | --- | --- |
| Maternal age, in years; mean±SD | 33.6±5.2 | 33.8±5.3 | 33.6±5.4 | 33.4±5.4 | 0.029 |
| Parity,^b^ no. (%) |  |  |  |  |  |
| Nulliparous | 2381.5 (37.3) | 2450.1 (39.8) | 2327.0 (36.4) | 2343.5 (36.5) | 0.077 |
| Parous | 3773.4 (59.1) | 3523.5 (57.2) | 3895.2 (60.9) | 3956.6 (61.6) |  |
| Missing | 231.3 (3.6) | 183.8 (3.0) | 172.7 (2.7) | 122.3 (1.9) |  |
| Race/ethnicity,^c^ no. (%) |  |  |  |  |  |
| White, non-Hispanic | 2146.4 (33.6) | 2019.2 (32.8) | 2176.5 (34.0) | 2254.5 (35.1) | 0.075 |
| Hispanic | 1890.7 (29.6) | 1884.0 (30.6) | 1797.5 (28.1) | 1692.3 (26.4) |  |
| Black, non-Hispanic | 973.4 (15.2) | 919.4 (14.9) | 986.5 (15.4) | 946.5 (14.7) |  |
| Asian | 1306.3 (20.5) | 1262.4 (20.5) | 1358.1 (21.2) | 1495.4 (23.3) |  |
| Other | 46.9 (0.7) | 41.0 (0.7) | 53.2 (0.8) | 7.1 (0.1) |  |
| Unknown | 22.5 (0.4) | 31.5 (0.5) | 23.1 (0.4) | 26.5 (0.4) |  |
| Maternal BMI,^b^ no. (%) |  |  |  |  |  |
| Normal or underweight (< 25 kg/m^2^) | 834.5 (13.1) | 832.6 (13.5) | 936.8 (14.6) | 960.7 (15.0) | 0.140 |
| Overweight (25-29.9 kg/m^2^) | 1422.8 (22.3) | 1224.4 (19.9) | 1466.1 (22.9) | 1306.7 (20.3) |  |
| Obese (≥ 30 kg/m^2^) | 3757.0 (58.8) | 3396.8 (55.2) | 3553.7 (55.6) | 3459.8 (53.9) |  |
| Missing | 371.8 (5.8) | 703.6 (11.4) | 438.3 (6.9) | 695.1 (10.8) |  |
| Tobacco use in pregnancy, no. (%) | 303.0 (4.7) | 274.1 (4.5) | 304.5 (4.8) | 399.3 (6.2) | 0.039 |
| Chronic hypertension,^c^ no. (%) | 5581.0 (87.4) | 5467.1 (88.8) | 5575.7 (87.2) | 5774.4 (89.9) | 0.050 |
| Pre-gestational diabetes,^c^ no. (%) | 1230.5 (19.3) | 1208.8 (19.6) | 1190.4 (18.6) | 1253.3 (19.5) | 0.014 |
| Education,^b^ no. (%) |  |  |  |  |  |
| Less than college | 3363.9 (52.7) | 3293.5 (53.5) | 3219.6 (50.3) | 3867.3 (60.2) | 0.112 |
| Completed college, or beyond | 2772.3 (43.4) | 2574.6 (41.8) | 2863.6 (44.8) | 2279.9 (35.5) |  |
| Missing | 249.9 (3.9) | 289.2 (4.7) | 311.6 (4.9) | 275.1 (4.3) |  |
| Timing of initiating prenatal care,^b^ no. (%) |  |  |  |  |  |
| First trimester^d^ | 5409.6 (84.7) | 4885.6 (79.3) | 5358.0 (83.8) | 5056.4 (78.7) | 0.107 |
| After first trimester | 406.4 (6.4) | 462.3 (7.5) | 449.2 (7.0) | 562.2 (8.8) |  |
| Missing | 570.2 (8.9) | 809.4 (13.1) | 587.6 (9.2) | 803.8 (12.5) |  |
| Weeks of gestation at index fill,^c^ mean±SD | 18.2±9.1 | 17.9±9.0 | 18.2±9.1 | 17.6±9.0 | 0.038 |
| Systolic BP before index fill, mm Hg; mean±SD | 140.0±17.6 | 139.8±16.0 | 141.9±17.4 | 141.1±19.1 | 0.071 |
| Diastolic BP before index fill, mm Hg; mean±SD | 86.0±12.5 | 85.8±11.0 | 87.5±13.0 | 85.8±11.2 | 0.071 |
| BP ≥ 160/110 before this pregnancy, no. (%) | 1882.2 (29.5) | 1809.9 (29.4) | 1962.4 (30.7) | 2013.8 (31.4) | 0.026 |
| BP ≥ 160/110 during pregnancy before the index fill, no. (%) | 1182.3 (18.5) | 1050.9 (17.1) | 1167.5 (18.3) | 1693.6 (26.4) | 0.115 |
| Prior antihypertensive medication use, no. (%) |  |  |  |  |  |
| Prior use, continuous | 2383.8 (37.3) | 2352.6 (38.2) | 2324.0 (36.3) | 2692.0 (41.9) | 0.074 |
| Prior use with a gap | 1880.7 (29.5) | 1867.0 (30.3) | 1993.2 (31.2) | 1937.1 (30.2) |  |
| No prior use | 2121.6 (33.2) | 1937.7 (31.5) | 2077.6 (32.5) | 1793.2 (27.9) |  |
| Delivery year, no. (%) |  |  |  |  |  |
| 2005-2008 | 1679.4 (26.3) | 1726.1 (28.0) | 1685.9 (26.4) | 1952.2 (30.4) | 0.117 |
| 2009-2010 | 1555.8 (24.4) | 1549.2 (25.2) | 1440.6 (22.5) | 1191.3 (18.5) |  |
| 2011-2012 | 1597.1 (25.0) | 1466.0 (23.8) | 1681.4 (26.3) | 1888.8 (29.4) |  |
| 2013-2014 | 1553.9 (24.3) | 1416.0 (23.0) | 1586.9 (24.8) | 1390.1 (21.6) |  |

Abbreviations: BMI, body mass index; BP, blood pressure; SD, standard deviation; SMD, standardized mean difference.

^a^All characteristics were measured prior to the index medication fill, except for delivery year. For this table, unstabilized weights were used.

^b^Covariates that are not in the propensity score model for treatment and thus are not expected to be balanced after weighting.

^c^Covariates that were included in the numerator used to calculate the stabilized weights.

^d^Defined as first 90 days of pregnancy.

# S6 Table. Distribution of analytic weights.

|  | Labetalol | Methyldopa | Other  β-Blockers | Nifedipine | All pregnancies |
| --- | --- | --- | --- | --- | --- |
| **Unstabilized treatment weights^a^** | | | | | |
| Mean (SD) | 2.12  (0.83) | 3.36  (1.81) | 16.47  (21.79) | 5.89  (3.63) | 4.00  (6.68) |
| Median (IQR) | 1.86  (1.59, 2.36) | 2.84  (2.17, 3.97) | 9.37  (4.01, 19.57) | 4.93  (3.48, 6.8) | 2.47  (1.82, 4.00) |
| Percent of weights greater than 10 (N) | 0  (0) | 0.01  (18) | 0.46  (182) | 0.10  (109) | 0.06  (309) |
| **Stabilized treatment weights^b^** | | | | | |
| Mean (SD) | 1.01  (0.35) | 0.99  (0.39) | 1.02  (0.85) | 1.01  (0.27) | 1.00  (0.40) |
| Median (IQR) | 0.91  (0.79, 1.11) | 0.87  (0.68, 1.22) | 0.83  (0.57,1.19) | 0.97  (0.82, 1.16) | 0.91  (0.76, 1.15) |
| Percent of weights greater than 10 (N) | 0 | 0 | 0.003  (1) | 0 | (1) |
| **Stabilized analytic weights including weights for missing information due to stillbirth^c^** | | | | | |
| Mean (SD) | 1.01  (0.34) | 0.99  (0.39) | 1.02  (0.86) | 1.01  (0.28) | 1.00  (0.40) |
| Median (IQR) | 0.90  (0.79, 1,11) | 0.87  (0.68, 1.21) | 0.82  (0.57, 1.19) | 0.97  (0.91,1.17) | 0.91  (0.76, 1.15) |
| Percent of weights greater than 10 (N) | 0 | 0 | 0.003  (1) | 0 | (1) |

Abbreviations: SD, standard deviation; IQR, interquartile range.

^a^Defined as one over the probability of observed treatment. For example, for pregnancies exposed to labetalol the unstabilized weight is defined as one over the probability of labetalol given covariates.

^b^Defined as probability of observed treatment given small set of covariates (defined in Methods) times unstabilized treatment weights. These analytic weights were used for all analyses to adjust for treatment selection confounding.

^c^Defined as stabilized treatment weights multiplied by stabilized weights for stillbirth/termination, to account for missing data because outcome information for these outcomes is undefined when a stillbirth or termination occurs: small for gestational age, preterm delivery, or neonatal ICU admission.
